# Evolution of the visual system in ray-finned fishes

**DOI:** 10.1017/S0952523823000020

**Published:** 2023-12-20

**Authors:** Michael H. Hofmann, Isabelle C. Gebhardt

**Affiliations:** Department of Comparative Neuroanatomy, Institute of Zoology, University of Bonn, Bonn, Germany

**Keywords:** ray-finned fishes, fovea, eye movements, visual processing, evolution

## Abstract

The vertebrate eye allows to capture an enormous amount of detail about the surrounding world which can only be exploited with sophisticated central information processing. Furthermore, vision is an active process due to head and eye movements that enables the animal to change the gaze and actively select objects to investigate in detail. The entire system requires a coordinated coevolution of its parts to work properly. Ray-finned fishes offer a unique opportunity to study the evolution of the visual system due to the high diversity in all of its parts. Here, we are bringing together information on retinal specializations (fovea), central visual centers (brain morphology studies), and eye movements in a large number of ray-finned fishes in a cladistic framework. The nucleus glomerulosus-inferior lobe system is well developed only in Acanthopterygii. A fovea, independent eye movements, and an enlargement of the nucleus glomerulosus-inferior lobe system coevolved at least five times independently within Acanthopterygii. This suggests that the nucleus glomerulosus-inferior lobe system is involved in advanced object recognition which is especially well developed in association with a fovea and independent eye movements. None of the non-Acanthopterygii have a fovea (except for some deep sea fish) or independent eye movements and they also lack important parts of the glomerulosus-inferior lobe system. This suggests that structures for advanced visual object recognition evolved within ray-finned fishes independent of the ones in tetrapods and non-ray-finned fishes as a result of a coevolution of retinal, central, and oculomotor structures.

## Introduction

The visual system consists of the eye with the retina and the accessory optic systems, the central visual information processing areas, and a motor system controlling eye movements. The evolution of the visual system can be the result of specializations achieved in these components independently, but frequently a coevolution is necessary to develop specific visual functionality. However, studies integrating the three components into a single evolutionary framework are lacking. Ray-finned fishes show enormous diversity and adaptations in all components of the visual system and are well suited for a cladistic analysis of visual evolution.

There is lot of information on retinal specializations, including visual pigments and color vision, spatial resolution, and retinal topography in a large number of species. Some excellent reviews and monographs were published, but only a few of them can be mentioned here (Walls, 1942; Ali & Anctil, 1976; Nicol, 1989; Collin & Shand, 2003). Regional specializations of the retina are known as ‘areas’ that show higher spatial resolution and may be organized into horizontal streaks or temporal areas that may facilitate the analysis of objects in overlapping receptive fields from both eyes. In some fish, small parts of the retina are histologically distinct and form a fovea with extra high spatial resolution, often in association with a high density of cones for color vision (Collin, 1999; Collin & Shand, 2003). This suggests a special role of the fovea in object identification.

Eye movements are less well studied on a comparative basis, but some species were investigated in more detail (Easter, 1971; Easter, 1972; Fernald, 1985; Fritsches & Marshall, 2002). Like in all vertebrates, the primary function of the eye muscles is to stabilize the image on the retina while the head or body moves. These smooth compensatory drifts of the eye relative to the head are usually not very obvious, but they are interrupted by saccades that occur in both eyes simultaneously to reset the eyes to a new position. Fish with retinal specialization in the form of an area or fovea can move their eyes independent of body movements to direct the area to objects of interest. These movements can be coordinated to direct the area or fovea onto an object in front for enhanced binocular vision. In fish with a distinct fovea, eyes can move quite independently to focus on objects of interest everywhere in the left or right visual field (Verrier, 1928, 1933; Kahmann, 1934; Walls, 1942; Schwassmann, 1968; Wagner et al., 1976; Easter, 1992; Pettigrew & Collin, 1995). These movements are typically independent and ‘chameleon-like’.

Central visual pathways are well investigated in a number of species. Major target of retinal ganglion cells is the mesencephalic tectum (Northcutt & Wullimann, 1988; Nieuwenhuys, 1998). Few fibers also innervate other diencephalic targets mostly in the pretectum. The tectum is connected with several accessory structures with additional functions. These are the torus longitudinalis (Wullimann, 1994; Ito et al., 2003), the nucleus isthmi (Xue et al., 2001; Northmore & Gallagher, 2003), and several pretectal areas (Braford & Northcutt, 1983; Striedter, 1990; Wullimann et al., 1991; Folgueira et al., 2008). Some of those are involved in the coordination of eye movements (Wang et al., 2020). There are also reciprocal connections with other sensory areas like the torus semicircularis that receives lateral line and auditory information from the brain stem (Schellart, 1983). Other more indirect pathways reach the telencephalon (Ito & Vanegas, 1983; Hagio et al., 2018). Another prominent pathway in many teleosts is a projection via the nucleus corticalis and nucleus glomerulosus to the inferior lobes (Wullimann & Meyer, 1990; Shimizu et al., 1999; Ahrens & Wullimann, 2002; Yang et al., 2007). The tectum has also prominent descending projections to the brain stem reticular formation to organize approach, orienting, and avoidance reactions (Niida et al., 1989; Pérez-Pérez et al., 2003; Gahtan et al., 2005; Barker & Baier, 2015).

We have investigated the brain organization in a large number of ray-finned fishes by measuring the sizes of many different areas and described the general diversity of the brains of ray-finned fishes elsewhere (Gebhardt & Hofmann, 2023). Here, we present data from more areas that are relevant for visual information processing and integrated them with published data on retinal specializations, particularly the presence of a fovea. Furthermore, we screened a large number of species for eye movements and produced the first comparative data set on the presence of independent eye movements in fish. Here, we bring all information together into a cladistic framework that gives new insights into the coevolution of eye, brain, and behavior in ray-finned fishes.

## Materials and methods

### Retinal specializations

Information about the presence of a fovea was taken from the literature. Since we are also interested in information about species that do not have a fovea, we searched specifically for keywords like retinal topography, photoreceptor and ganglion cell distribution, ‘area’, and ‘horizontal streak’. In addition, we made an automated CrossRef search for all taxon names of ray-finned fishes and collected more than 200,000 references. Titles and abstracts were screened for retina and full texts were downloaded wherever possible. This allowed also high-speed customized full-text searches. We could identify a total of 203 species with information about the retinal topography and in 57 of them a fovea have been described (see Supplementary Material). There were many more species with descriptions of the photoreceptor arrangements and other histological details of the retina, but if there were no explicit documentation of the retinal topography, the studies were not included here.

### Eye movements

Eye movements were classified by inspection of freely moving animals in public aquaria, mainly Aquazoo Düsseldorf and Köln Zoo, and at local dealers. Some species were available at YouTube. Most species show eye movements associated with body turns. They occur in both eyes at the same time, one eye moves forward, the other backwards. They are not always associated with locomotion, sometimes only a small change in body orientation or just a tail bending was observed. Independent eye movements were classified as movements that occur in a single eye and were not timed to a movement of the other eye. Eye movements were evaluated only if both eyes are visible to the observer. Attention is given to the eye that is less visible. Any movement detected there can be compared to the movement of the more visible eye. The more visible eye gives a good movement signal even if that is not in the center of the eye of the observer. Some species do not move much during food search like Syngnathidae, but others are steadily swimming (Tetraodontiformes). Wrasses are usually very active during food search. Here, eye movements can be inspected only if the fish are slowing down and inspect objects locally. If the observer cannot clearly see independent eye movements in a few minutes, the species was skipped.

Since we want to make a direct correlation between eye movements and brain morphology in the same species, we were focusing on the very species where we had brain data. Sometimes, several individuals of the same species were in the same aquarium and we monitored the one that is in the best view, often having to switch from one individual to the other. So it is impossible to give a number of individuals per species that we tracked, and each individual was tracked for variable times. It was also not possible to use eye tracking equipment as handling and interference with the animals would require animal protocols for all 168 species. However, if a species had independent eye movements, it was always quite obvious and easy to see.

### Volume measurements

Volumes of brain parts were measured based on image stacks created by block face imaging. The procedure was described in Gebhardt and Hofmann (2023) in more detail. In brief, fish were anesthetized, the brains exposed, and fixed by immersion in 4% formalin. The brains were removed a day later and stored in fixative for at least another day. Before staining and embedding, brains were transferred to 70% ethanol for three days, followed by 0.5% cresyl violet in distilled water for three hours. After washing briefly with distilled water, brains were dehydrated in ethanol and embedded in methacrylate (Technovit 7100, Kulzer, Germany) according to the supplier’s protocol. Then, blocks were trimmed and cut on a custom microtome equipped with a custom-built fluorescence microscope to image the block surface after each section. Section thickness was 5 μm and image resolution 4 MP. Objective magnification was varied according to the size of the brain.

**Figure 1.**
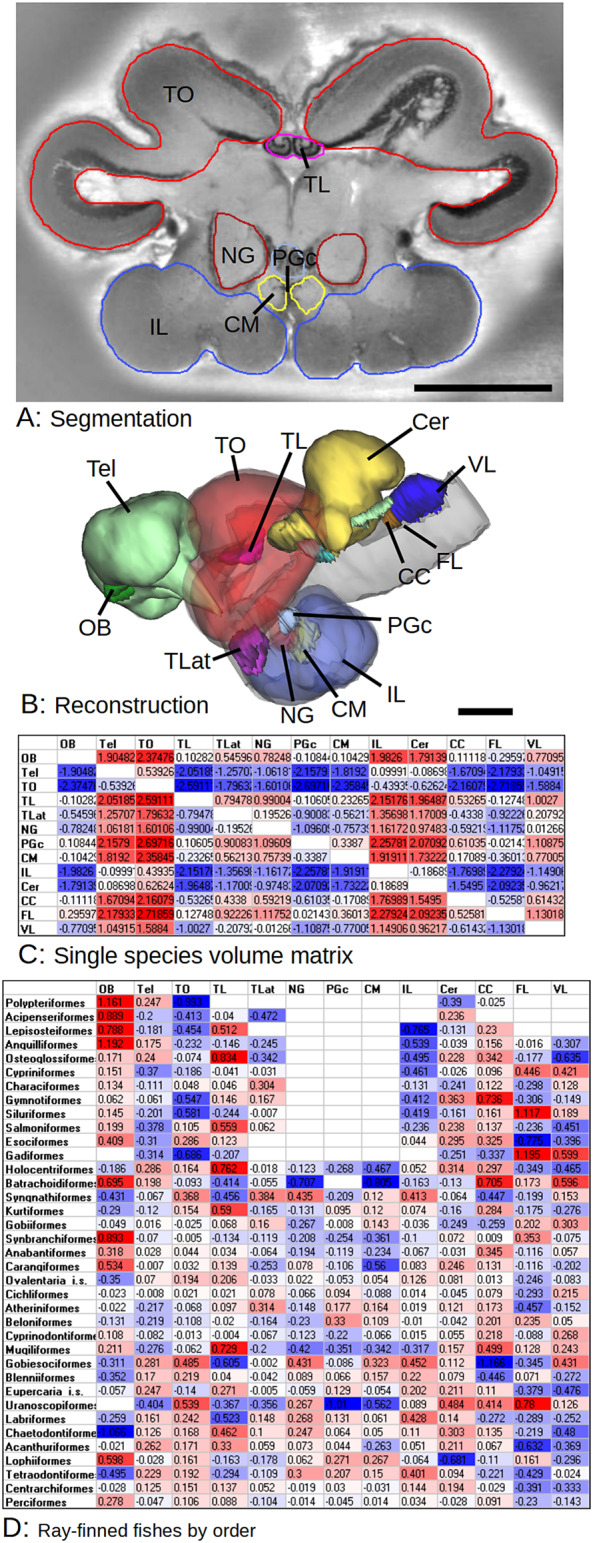
Brain part segmentation and the construction of a volume ratio matrix. (A) Section through the midbrain of *Synchiropus ocellatus* with the contours of different brain parts. (B) 3D reconstruction of all brain parts used in this study. (C) Volume ratio matrix. Each cell in the matrix is the ratio of the part in each the column divided by the part in each row, log10 transformed. The volume ratio matrix is independent of any absolute scaling and is the basis of other calculation. (D) Summary of relative brain part volumes averaged by order. The total average of all species was calculated and the individual order values subtracted and log-transformed. Red color codes indicate the part is larger than average, blue color codes means the part is smaller. Abbreviations: CC, crista cerebellaris; Cer, cerebellum; CM, corpus mamillare; FL, facial lobe; IL, inferior lobe; NG, nulceus glomerulosus; OB, olfactory bulb; PGc, commissural preglomerular nucleus; Tel, telencephalon; TL, torus longitudinalis; TLat, torus lateralis; TO, tectum opticum; VL, vagal lobe. Scale bars equal 1 mm.

Volume measurements were done with a custom program. Brain parts were segmented manually in the image stacks and the program calculated the absolute volume in mm^3^. We are using here data already published in Gebhardt and Hofmann (2023). Some additional areas were measured for this study. These are the nucleus glomerulosus pars rotundus (NG), corpus mamillare (CM), and the commissural preglomerular nucleus (PGc). Figure 1 shows an example image with the segmentation of these areas. For the definition of the other areas see Gebhardt and Hofmann (2023). A list of all brain areas measured in this study is shown in Table 1.

**Table 1.** List of all brain areas measured in this study

CC	Crista cerebellaris
Cer	Cerebellum
CM	Corpus mamillare
FL	Facial lobe
IL	Inferior lobe
NG	Nucleus glomerulosus
OB	Olfactory bulb
PGc	Nucleus preglomerulosus commissuralis
Tel	Telencephalon
TL	Torus longitudinalis
TLat	Torus lateralis
TO	Tectum opticum
VL	Vagal lobe

A total of 168 species were used in this study for volume measurements. To compare brain part volumes between species with different overall sizes, relative volumes were calculated. Instead of using a reference volume (total brain), we were creating a ratio matrix for each animal by dividing the volume of each brain part by each other as described in Hofmann (2020). Each cell in the matrix is a ratio of two brain parts; all scaling differences between animals are thus removed. The matrices can now be used to calculate averages of all animals, or the animals can be grouped and the group averages compared by subtracting them to obtain a difference matrix.

### Phylogenetic framework

For a phylogenetic analysis, we used a time-calibrated phylogenetic tree for ray-finned fishes from Rabosky et al. (2018). The tree data were imported in R-Studio and pruned to the species with brain data and the species list compiled from our literature survey of the presence of the fovea.

### Data analysis

We used R-Studio version 2023.06.1 for further data processing. After importing the phylogenetic tree and the traits, we calculated the phylogenetic signal using the function phylosig from the package phytools (Revell, 2012). The phylogenetically independent contrasts of the brain part volumes were calculated with the get_independent_contrast method of the castor package (Louca & Doebeli, 2018). Correlation matrices were calculated with the rcorr function of the Hmisc package. The cluster analysis of the brain areas was done with the pvclust package. For the principle component analysis we used the prcomp function for the original values and the ppca function from the adephylo package to calculate phylogenetically independent principle components (ppca, Jombart et al., 2010). For the cluster analysis and the ppca, it was necessary to replace some missing values. Some brain parts were not measurable and the missing values for that species replaced with the one of the closest relative based on the distances in the tree.

### Related publication

A subset of the data presented here was published by Schmidt (2020) without the current authors’ knowledge. This makes it necessary to comment on Schmidt’s contributions to the entire project. Schmidt was not involved in the planning or designing of the 2020 project, or in the creation of the image stacks presented in that paper. That paper presents segmented volume data that were part of the thesis of one of us (Gebhardt, 2022) and another publication (Gebhardt & Hofmann, 2023) without mentioning the source of the data. The data as presented in the present paper form the authoritative record of discovery.

## Results

### Retinal specializations and eye movements

The retinal topography has been studied in a large number of species. In many cases, a topographic map of ganglion cell density or photoreceptor density was constructed. In other studies, regional differences were noted only in the text. Here, we present only information about the presence of a fovea.

Eye movements have been described in the literature in a few species only. To obtain a species list that can be correlated with our brain volume data, the same species has to be screened. If that was not possible, at least the same genus was investigated. We present here only data on the presence of independent eye movements. Independent means here temporally independent, that is, saccades in one eye are not correlated with a saccade in the other eye as described in material and methods.

Table 2 shows the combined results of the literature search on the presence of a fovea and our survey on independent eye movements at a family level. It should be noted that there are only a few species studied within a family, sometimes only one. The table helps to list the incidences in a systematic way but does not mean that all members of the family are alike. A detailed species list if available in Supplementary Material.

**Table 2.** List of all families with information about the presence of a fovea and independent eye movements (iEye)

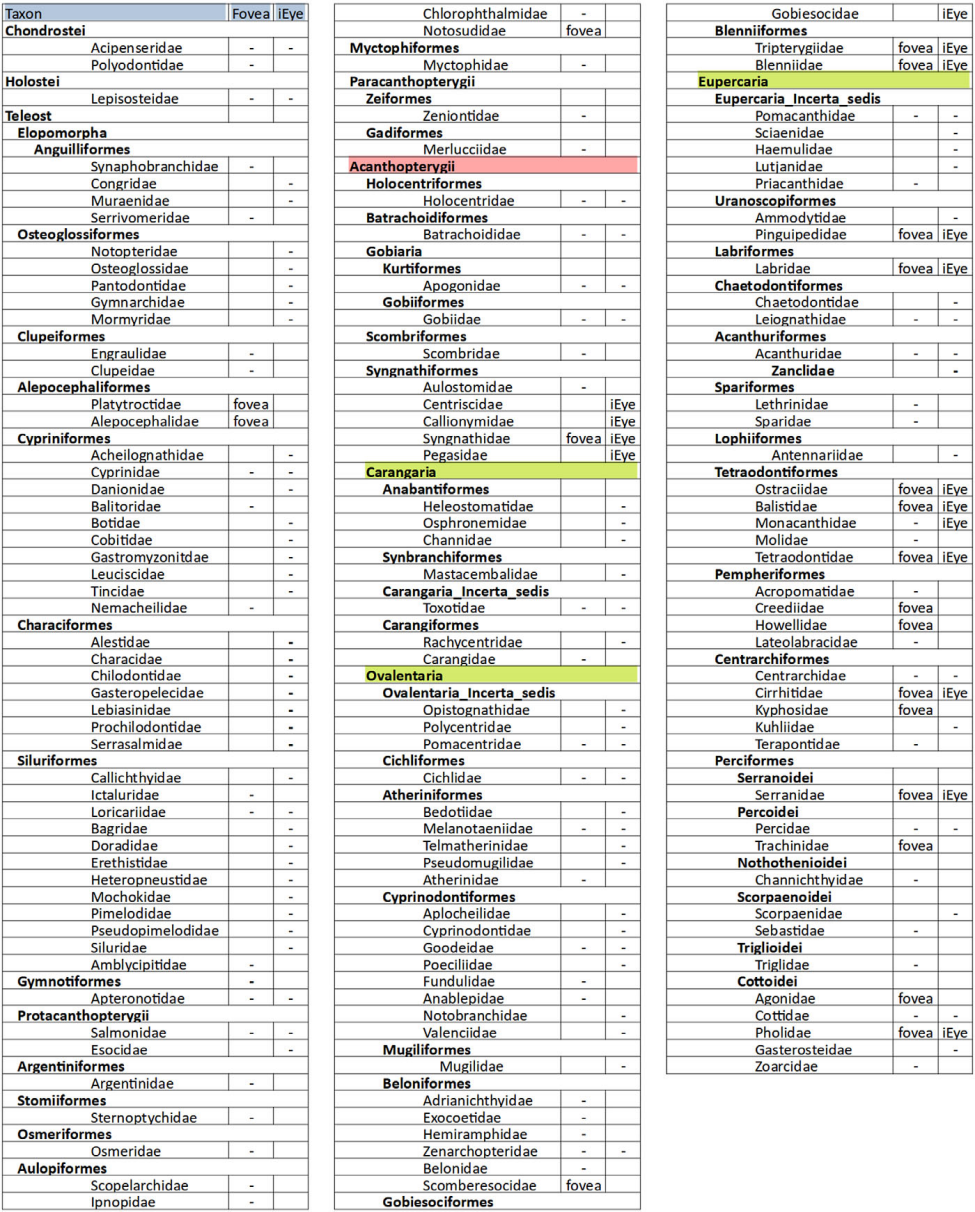

*Note:* A dash represents families with at least one species described without a fovea or independent eye movements. Empty spaces indicate that there is no information available in that family. See Supplementary Material for a complete list of all species.

A fovea and independent eye movements could not be found in any taxa outside the Acanthopterygii except for the Alepocephalidae and the Notosudidae, which are all deep-sea species. The fovea is well investigated, but there are no information on eye movements or detailed brain morphology. Within Acanthopterygii, the first group showing well-developed fovea and extreme mobile eyes are the Syngnathiformes (sea horses, sea needles, and dragonets). Next groups are the Blenniiformes and Gobiesociformes. Blennies are long known for their fovea and independent eye movements but there is no information on the retina in clingfish (Gobiesociformes). We studied only one species (*Diademichthys lineatus*) and it had extremely mobile, independent eye movements.

The next group where we have brain volume data are the wrasses (Labridae). The foveae of wrasses are well investigated in a number of species. However, the situation in *Labroides dimidiatus* is unclear. This is the cleaner wrasse. Although we have brain data, we could not get information about eye movements or a fovea. Another group with extreme mobile eyes and a fovea are the Tetraodontiformes. However, not all species in this group have a fovea (e.g., some Monacanthidae and Molidae lack it).

The last orders in the table all have species that have a fovea and also independent eye movements in some of their families. The most important family here are the Cirrhitidae because we have brain data of some species, and all of them were available to inspect the eye movements.

### Volumetric measurements

Volumes of different brain parts were measured in more than 160 species from all major groups of actinopterygian fishes. However, we were restricted to commonly available species and some interesting groups like deep sea fish and pelagic marine species were not available. Some data used here were already published in Gebhardt and Hofmann (2023). Additional data were measured for the NG, PGc, and CM. Figure 1 shows an example section with the segmented brain parts and a 3D reconstruction of the brain of *Synchiropus ocellatus.* Figure 1A,D shows the volume data in a table form for all species investigated at the order level. All species within an order were averaged and the difference to the total average of all actinopterygians calculated for each order. Shades of red indicate brain parts bigger than the average in the order and shades of blue parts that are smaller than the average. Color codes are centered around white, meaning the value is equal to the average.

### Cladistic analysis

**Figure 2.**
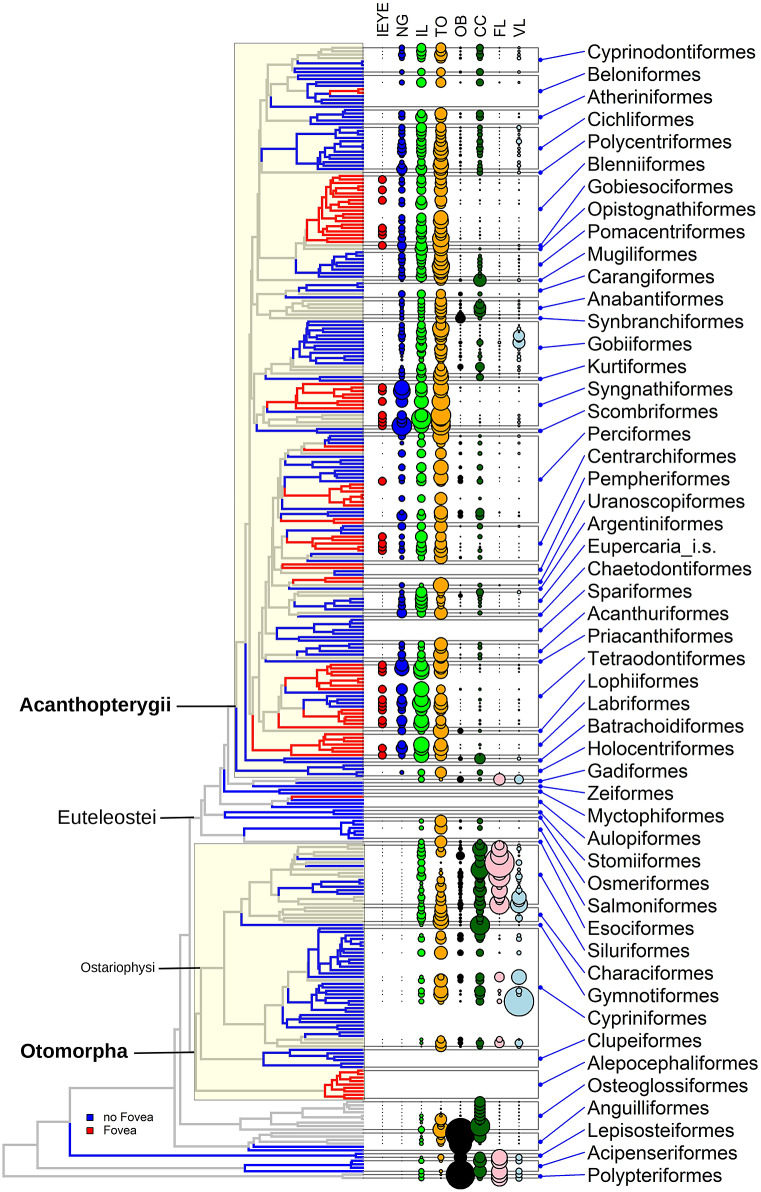
Phylogenetic tree of all species used in this study. The tree branches are color coded for the presence of a fovea (at a family level) according to our literature search. Circles represent the traits measured in this study. The first column of red dots indicate the presence of independent eye movement. The other columns are showing the relative size of different brain areas.

Simply looking at two groups is ignoring the complex phylogenetic relationships of the data points (species). For a cladistic analysis, we were using a time-calibrated phylogenetic tree (Rabosky et al., 2018) with several traits plotted at the tips (Fig. 2). The tree branches were colored according to the literature data on the presence of a fovea, generalized to the family level. That means that families with at least one species with a fovea are colored red, families with no fovea are colored in blue and families without data are gray. Next to the tips, we plotted the presence of independent eye movements according to our survey with red dots. The other dot plots indicate the relative size of different brain parts, after scaling the original values (minus mean divided by standard deviation).

A fovea and independent eye movements are found only in Acanthopterygii (Fig. 2). One exception is the Alepocephaliformes. They are deep sea fish and all investigated species show a distinct fovea. Unfortunately, they were not available to check for eye movements or a brain part analysis. The same is true for other deep sea fish like the Notosudidae which also have a fovea. An NG is also only present in Acanthopterygii. Its size correlates with independent eye movements and the presence of a fovea. The IL is present also in non-Acanthopterygii, but it is much smaller (Fig. 2). In Acanthopterygii, the size of the IL seems to correlate with the size of the NG. The TO does not show a consistent pattern. The other brain areas plotted represent different non-visual areas. The sizes vary tremendously, but the largest values are found always among non-AcanthopterygiiSome of these observations were quantified in a phylogenetic context in the next sections.

### Brain area correlations

**Figure 3.**
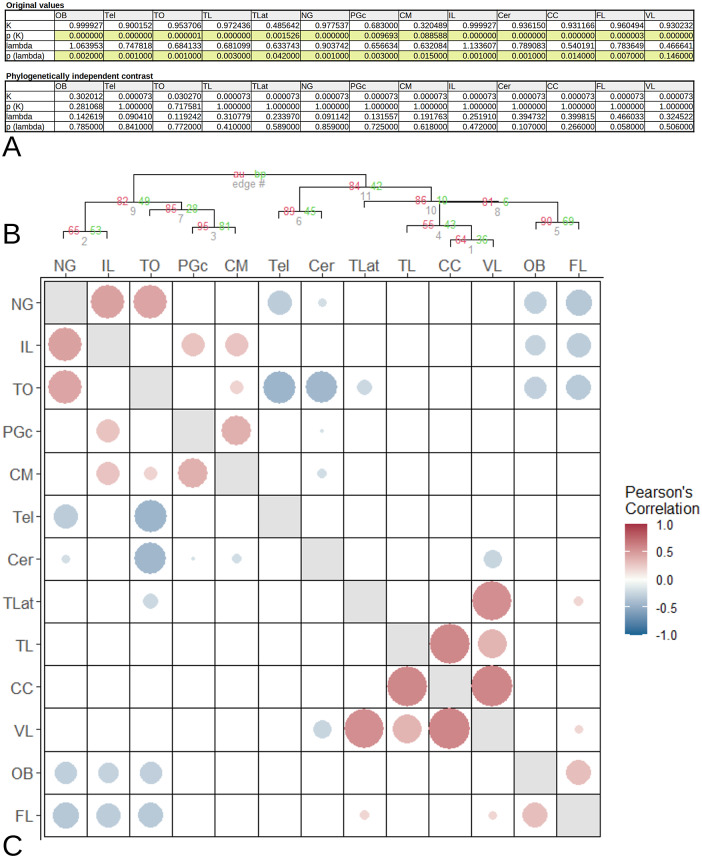
Phylogenetic analysis of brain part volumes. (A) Phylogenetic signals of the brain areas. The top table shows the *K*- and lambda values and their *p*-values of the original brain volumes. There is a strong phylogenetic signal in all brain parts (*p* < 0.05, green color code). After calculating the phylogenetically independent contrasts (PICs), the phylogenetic signal was completely eliminated (lower table in A). (B) Dendrogram showing the results of the hierarchical cluster analysis of the PICs. The red values represent the probability. Values above 95 are equivalent to a *p*-value of <0.05. The clustering results are not very reliable since most values are below 95. However, the dendrogram is used to sort the correlation matrix in (D). The matrix shows the Pearson correlation coefficients of all brain part pairs. Size and color of the dots represent the correlation coefficient. Dots are only shown for correlations with *p*-values <0.05. There is a strong correlation cluster among NG, IL, and TO and another one among TLat, TL, CC, and VL.

Since we are particularly interested in the NG and IL and associated CM and PGc, we restricted our analysis to Acanthopterygii because these areas are missing in more basal clades. We first tested whether the original brain volume data have a phylogenetic signal. Figure 3A shows that all have a strong phylogentic component based on their K and lambda values. We then calculated the phyogenetically independent contrast (PIC). We then rechecked the phylogenetic signal of the PICs. As expected, the K and lambda values of the PICs were all non-significant indicating that the phylogenetic signal was effectively removed. In R-Studio, we created a cluster dendrogram of the brain areas (Fig. 3B) based on their PIC values. The dendrogram is not very robust based on the low probabiliy values of their branches. It is only used to sort the correlation matrix. The correlation matrix was created based on the Pearson correlation coefficient. Figure 3C shows a bubble plot of the correlations. Only the significant (*p* < 0.05) data were plotted This showed that there is a strong correlation between NG and IL and TO, and a weaker correlation with CM and PGc. Another strong correlation cluster is present among TLat, TL, CC, and VL. The NG, IL, and TO cluster shows a negative correlation with OB and FL.

### Principle component analysis

**Figure 4.**
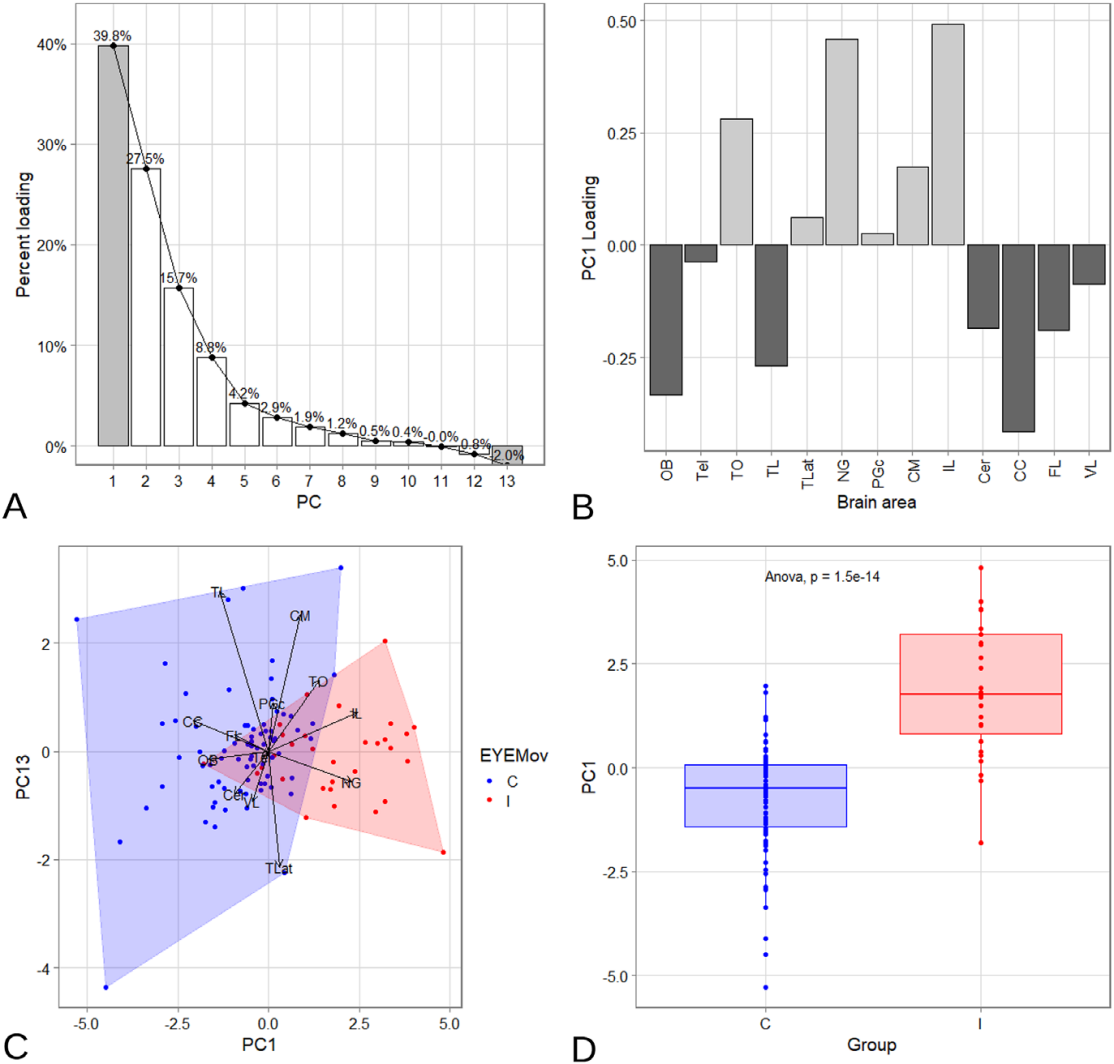
Phylogenetic Principal component analysis within Acanthopterygii. (A) Bar graph showing the relative loadings of different components. (B) The contribution of the brain parts to the first component (PC1). NG, IL, and TO dominate the PC1. (C) Scatter plot of all Acanthopterygii species for the first and 13th principal component. The vectors showing the contributions of all brain areas. Species are color coded. Red color represent the species with independent eye movements (group ‘I’) and blue indicates species with only compensatory eye movements (group ‘C’). (D) Box plot and ANOVA analysis of the scores of PC1 showing a significant separation between both groups.

Next, we were analyzing the patterns of brain areas volumes with a phylogenetic principle component analysis (pPCA). Figure 4A shows the loadings of the principal components and Fig. 4B the contribution of the brain areas to the first component (PC1). This confirmed the correlation analysis because NG, IL, and TO contributed to a high degree to PC1. The negative components correspond to the correlation cluster found in the correlation matrix consiting of CC, VL, TL, and FL. The loading of the PC1 in the pPCA showed that both correlation clusters are anti-correlated in the samples meaning that each species tends to show either one or the other cluster. Figure 4C shows a scatter plot of the species for the PC1 and PC13 and the loading vectors for all brain areas. The species are color coded. Red means they have independent eye movements and the species colored in blue have only compensatory eye movements according to our classification. The scatter plot shows a clear separation in the PC1 axis. This was confirmed by an ANOVA (Fig. 4D) showing a significant separation caused by PC1. Since the PC1 contains mostly NG, IL, and TO, it can be concluded that those areas are correlated with independent eye movements.

## Discussion

The visual system consists of the eyes, central information processing areas, and an oculomotor system. In a more basic scenario, the outside world is projected onto the retina and the oculomotor system is stabilizing this image during head or body movements. Any changes can then be analyzed by central information processing with movements being the most simple to compute. This system can be elaborated in different ways. Enhanced resolution and color vision can be used to detect finer details in objects, allowing for object classification that is useful for prey selection or establishing more complex social interaction with identifiable conspecifics. On the other hand, the relative location of objects and relative movements to each other can be used for spatial orientation and place memory. In primates, where both systems are highly elaborated, visual information processing is progressively channeled from the primary visual cortex into two separate paths, specialized in object recognition and object location and movement, respectively (Kandel, 2013, Chapter 28). This is accompanied by the development of a fovea (Baden et al., 2019) and non-compensatory eye movements like voluntary saccades and pursuit eye movements (Schutz et al., 2011).

The elaboration of the visual system is, like many other systems, adaptive. Certain life styles and habitats require the evolution of more complex visual systems and other sensory mechanism may be better suited to other situations. We see that within mammals (Hughes, 1977), but also within any other animal group. To facilitate a broader comparative study in ray-finned fishes, we have to rely on easy accessible features and were choosing here information on the presence of a fovea and independent eye movement. In addition, we were identifying and measuring many brain areas for signs of any elaboration of the central visual system and can now combine this information into a cladistic framework.

There are many visually related systems that would be interesting to investigate in an evolutionary context like the tectal organization, nucleus isthmi, pretectum/accessory optic system, and the preglomerular areas relaying visual information to the telencephalon. Here, we focus on the pathway from the tectum via the nucleus corticalis and nucleus glomerulosus to the inferior lobes.

### Brain organization

First, we wanted to see whether there are any consistent features in brain morphology that correlate with the presence of a fovea or independent eye movements. Literature data on the fovea are available for many species, but they rarely match the species where we have brain data. The presence of a fovea, however, is tightly correlated with the presence of independent eye movements. So we screened a large number of species for independent eye movement including the ones where we have brain data. If the exact species is not available, we investigated members of the same genus.

Comparing brain anatomy with the presence of independent eye movements shows that there is a high correlation with the size of the IL and NG in Acanthopterygii. The TO is also correlated, but to a lesser degree. There is also some correlation between the CM and PGc. The fact that there is a better correlation with the NG and IL than with the TO, which gets direct retinal input, is interesting. In foveate fishes, the area in the TO that represents the fovea is enlarged (Schwassmann, 1968), but the correlation is better with NG and IL that probably serve a more specific visual function in object recognition. The Pearson correlation analysis shows that the NG and IL are highly correlated, in fact size correlation is one of the highest of all possible part combination in the brain. This suggests that NG and IL form a functional unit which is also supported by the anatomical data described next.

The NG of Acanthopterygii consists of a small anterior part and a caudal pars rotundus (Ito & Kishida, 1975; Gómez-Segade & Anadón, 1988). The pars rotundus is very prominent and this is the part measured in this study and the abbreviation NG stands here for the pars rotundus only. It receives input from the tectum via the nucleus corticalis (NC) (Wullimann & Meyer, 1990; Shimizu et al., 1999; Ahrens & Wullimann, 2002; Yang et al., 2007). The NC consists of large cells that have extensive dendrites in tectal layers. NC cells respond to small moving objects and have very wide receptive fields (Rowe & Beauchamp, 1982). Their axons terminate in the NG and form distinct glomeruli. They have also collaterals reaching the contralateral NG via the horizontal commissure. The NG, in turn projects to the inferior lobe. Recordings from NG cells were done only in slices without natural stimuli so the function of the pathway is still uncertain (Tsutsui et al., 2001). The inferior lobes are located lateral to the hypothalamus, around a lateral ventricular recess that is originating in the third ventricle at the base of the hypothalamus. For that reason it is often considered to be part of the hypothalamus. However, the connections of the IL are quite different from the traditional hypothalamus and a study in zebrafish showed that most IL neurons are of mesencephalic origin rather than being part of the diencephalon as the medial hypothalamus (Bloch et al., 2019). The IL can be quite large, in some species like S*ynchiropus* or *Eurypegasus* the IL is even larger than the entire telencephalon (Gebhardt & Hofmann, 2023). The large size of the IL in wrasses has been noted also by Estienne et al. (2023). They concluded that the NG-IL system is a derived feature in ray-finned fishes and has no counterpart in tetrapods. Furthermore, a recent study using neuronal activity marker could show that the IL is involved in visual learning (Calvo et al., 2023). This study, the anatomy, the physiology of the NC cells, and the size correlation with the presence of a fovea reported here strongly suggests that this pathway is a functional unit (the NG-IL system) and is involved in visual object identification.

### Evolutionary considerations

There are two major groups of ray-finned fishes. One group consists of cyprinids, silurids, and characids. Together with the clupeomorphs and alepocephaliforms they form the Otomorpha. The sister group of the Otomorpha are the Euteleostei. The Euteleostei form a number of smaller groups but one group, the Acanthopterygii, have by far the greatest number of species. The Acanthopterygii and the Otomorpha comprise both about a third of the total ray-finned fish species and together about two-thirds of all. There are a number of important morphological differences between Otomorpha and Acanthopterygii that lead to distinct ecological niches they occupy. Differences in their visual system may have contributed to the way they made their journey through evolution.

Otomorpha have also an IL, but it is smaller and a distinct NG pars rotundus is missing (Ito & Kishida, 1975; Wullimann & Meyer, 1990; Butler et al., 1991). It is interesting to note that there is no report of a fovea in basal ray-finned fishes and we did not find any species with independent eye movements. This would suggest that object recognition is less advanced in Otomorpha. The majority of Otomorpha are Ostariophysi with three major groups, cypriniforms, characiforms, and siluriforms. They dominate in most freshwater environments and are very divers and successful. Our previous study showed that these freshwater habitats favor non-visual senses like chemoreception and lateral line (Gebhardt & Hofmann, 2023). They are also hearing specialists due to a unique connection of the swim bladder with the inner ear (Chardon & Vandewalle, 1996). Many groups are also known to communicate with sound (Parmentier & Diogo, 2006; Bass et al., 2015; Rice et al., 2022). In addition, all catfish have passive electroreceptors (Finger, 1986; Peters, 2008) and have also an extensive taste system covering the entire body surface (Finger, 1976). The taste pathways are known to reach the IL via the secondary gustatory nucleus (Kanwal et al., 1988). These non-visual senses are certainly very important for object recognition and communication. The message here may be that Ostariophysi do not lack an elaborated visual object recognition pathway because they are ‘basal’, but because other senses are better suited for object recognition in their freshwater habitats. Our results presented in Fig. 3 showed that non-visual centers are cleary better developed in Otomorpha and other more basal groups than in Acanthopterygii.

The distinction between freshwater and marine habitats is a simplification and not all marine habitats favour vision and not all freshater habitats other senses. Coastal lagoons may feature conditions like freshwater lakes and visual orientation may be limited in open oceanic waters. Even in coral reefs, there is a clear difference of the dominant species between day and night with visual specialists resting or hiding during the night. There are some non-acanthopterygian groups (without an NG) that are mostly marine. Clupeomorphs are chiefly migrating in open waters and many Anguilliforms are active at night in coral reefs or found in deeper waters. There are always some exceptions. Elopomorphs like ladyfish and tarpons are active visual hunters, but do not have a NG. Among Anguilliformes, the garden eels have a life style very similar to the acanthopterygian *Opistognathus.* We checked both for eye movements, but we could not classify them as having independent eye movements. Both species have burrows and head out into the current to catch plankton particles. This is similar to sea horses, which do have very pronounced independent eye movements. There are more examples of fish from very different groups that have similar habitats and life styles. These are interesting model systems to study how different animals from different phylogenetic backgrounds solve the same problem.

There are many deep-sea fishes among many major teleost groups. Their eyes show an enormous diversity including highly developed foveae in some groups (Locket, 1977; Collin & Partridge, 1996; Warrant et al., 2003; Warrant & Locket, 2004; de Busserolles et al., 2020). The alepocephalimorphs are a sister group of the Otophysi and all of them have a well-developed fovea (Collin & Partridge, 1996; Collin et al., 2000). The visual world in the deep sea is different from shallow water habitats. At greater depths, important light sources are bioluminescent organisms and, at a distance, can be considered point sources (Collin & Partridge, 1996). In contrast to the shallow water fish that usually have a pure cone fovea for color vision, the fovea of deep sea fishes is often characterized by high concentration of rods (Collin, 1999). The absence of color vision in the fovea of deep sea fishes suggests that the fovea may be used primarily to detect weak point sources rather than to analyze object properties.

More shallow marine habitats are dominated by the Acanthopterygii. These habitats are very diverse, but they include areas with high species density, complex environments, and good visibility. This is not only limited to coral reefs, but include also rocky shores, kelp forests, and other shallow water niches. Here vision may be superior for object discrimination than other senses. These are also the habitats where we found most of the foveate species correlated with an elaborate NG-IL system.

Some of the Acanthopterygii did invade other habitats. Several groups were moving back into fresh water. The major groups to mention here are the cichlids, cyprinidontiforms, anabantiforms, and atheriniforms, and they all have inherited the NG-IL system. Now they have to compete with the Otomorpha. With their ‘superior’ visual system, did they replace all the Otomorpha? The answer, of cause, is no. The freshwater Acanthopterygii are only successful in some habitats where they can play out their advantages. Examples are the cichlids in the great lakes in Africa, but also in some South American habitats. In Asia, east of India, the atheriniforms seemed to be more successful. Cyprinidontiforms and anabantiforms usually occupy smaller water bodies with complex visual structures, but they are limited to tropical or subtropical regions. There are large freshwater habitats that are still dominated by the Otomorpha. These may be the habitats where non-visual senses are more important. Of course, Acanthopterygii have evolved many other characters related to locomotion, feeding, fin positions, and spiny fin rays. It is impossible to pinpoint the exact features that are important for the competition with Otomorpha in different habitats, but advanced visual object recognition may be one of them.

As fascinating as it can be to speculate about visual adaptations and their impact on species diversity and distribution, the only thing we found here in this study is a correlation of the size of some brain structures with the presence of a fovea and independent eye movements. More specific, we found evidence that particularly the NG-IL system is larger in species that are specialized in object identification. The extreme size of the NG-IL system in foveate species, that are top specialists in object recognition, supports this view. The cladistic analysis shows that this system evolved within ray-finned fishes and the anatomy indicated that it evolved in areas completely different from higher visual areas in tetrapods. More sophisticated visual information processing thus evolved in fishes independent from tetrapods. Although most brain functions are probably highly conserved, others may be uniquely derived in different vertebrate groups even if they serve the same functions.

## Supporting information

Hofmann and Gebhardt supplementary material Hofmann and Gebhardt supplementary material

## Supplementary material

The supplementary material for this article can be found at http://doi.org/10.1017/S0952523823000020.
