# Supplementary material for: Evolution of the visual system in ray-finned fishes
Source: Vis Neurosci. 2023 Dec 20;40:E005. doi: 10.1017/S0952523823000020 (PMC11016354; doi:10.1017/S0952523823000020)
Supplement: Hofmann and Gebhardt supplementary material [file S0952523823000020sup001.pdf]

Supplementary Materials to:

Evolution of the Visual System in Ray-Finned Fishes

Hofmann, M.H. and Gebhardt, I.C.

Department of Comparative Neuroanatomy
Institute of Zoology
University of Bonn, Germany

Visual Neuroscience

Contents:

Table 1: List of species with independent eye movements

Table 2: List of species with retinal specializations

References to Table 2

Suppl. Table 1: Presence of eye movements as classified by our survey in public aquaria, fish dealer, and videos.

Species	Eye movements
**Actinopterygii**	
**Lepisosteiformes**	
**Lepisosteidae**	
Lepisosteus platostomus	conjugated
**Teleostei**	
**Elopomorpha**	
**Anguilliformes**	
**Congridae**	
Conger cinereus	conjugated
**Muraenidae**	
Echidna nebulosa	conjugated
Rhinomuraena quaesita	conjugated
**Ophichthidae**	
Myrichthys maculosus	conjugated
**Osteoglossomorpha**	
**Osteoglossiformes**	
**Gymnarchidae**	
Gymnarchus niloticus	conjugated
**Mormyridae**	
Brienomyrus brachyistius	conjugated
Brienomyrus niger	conjugated
Gnathonemus petersii	conjugated
Mormyrus longirostris	conjugated
**Notopteridae**	
Chitala chitala	conjugated
Xenomystus nigri	conjugated
**Osteoglossidae**	
Osteoglossum bicirrhosum	conjugated
**Pantodontidae**	
Pantodon buchholzi	conjugated
**Otomorpha**	
**Cypriniformes**	
**Gyrinocheiloidei**	
**Gyrinocheilidae**	
Gyrinocheilus aymonieri	conjugated
**Cobitoidei**	
**Cobitidae**	
Acantopsis choirorhynchos	conjugated
**Gastromyzontidae**	
Gastromyzon punctulatus	conjugated
**Cyprinoidei**	
**Acheilognathidae**	
Rhodeus amarus	conjugated
**Cyprinidae**	
**Barbinae**	
Puntius denisonii	conjugated
Systomus rhomboocellatus	conjugated
**Cyprininae**	
Carassius auratus	conjugated
**Leuciscidae**	
Chondrostoma nasus	conjugated
Phreatichthys andruzzii	conjugated
Rutilus rutilus	conjugated
Scardinius erythrophthalmus	conjugated
**Tincidae**	

Tinca tinca	conjugated
Characiformes	
**Characoidei**	
**Characidae**	
Aphyocharax anisitsi	conjugated
Hyphessobrycon pulchripinnis	conjugated
Inpaichthys kerri	conjugated
**Chilodontidae**	
Chilodus punctatus	conjugated
**Lebiasinidae**	
Nannostomus beckfordi	conjugated
Gymnotiformes	
**Sternopygoidei**	
**Apteronotidae**	
Apteronotus albifrons	conjugated
Siluriformes	
**Loricarioidei**	
**Callichthyidae**	
Corydoras reticulatus	conjugated
Megalechis thoracata	conjugated
**Loricariidae**	
Glyptoperichthys joselimaianus	conjugated
Macrotocinclus affinis	conjugated
Pterygoplichthys pardalis	conjugated
Rineloricaria parva	conjugated
Sturisoma aureum	conjugated
**Siluroidei**	
**Bagridae**	
Mystus bimaculatus	conjugated
**Doradidae**	
Platydoras costatus	conjugated
**Erethistidae**	
Hara jerdoni	conjugated
**Heteropneustidae**	
Heteropneustes fossilis	conjugated
**Mochokidae**	
Synodontis nigriventris	conjugated
Synodontis petricola	conjugated
**Pimelodidae**	
Zungaro zungaro	conjugated
**Pseudopimelodidae**	
Microglanis iheringi	conjugated
**Euteleostei**	
**Protacanthopterygii**	
Salmoniformes	
**Salmonidae**	
Oncorhynchus mykiss	conjugated
Salmo trutta	conjugated
Esociformes	
**Esocidae**	
Esox lucius	conjugated
**Acanthopterygii**	
Holocentriformes	
**Holocentridae**	
Myripristis murdjan	conjugated
Batrachoidiformes	
**Batrachoididae**	
Porichthys notatus	conjugated
**Scombrimorpharia**	

Syngnathiformes	
**Callionymoidei**	
Callionymidae	
Callionymus maculatus	independent
Synchiropus ocellatus	independent
Synchiropus splendidus	independent
**Syngnathoidei**	
Syngnathidae	
Corythoichthys haematopterus	independent
Dunckerocampus dactyliophorus	
Nerophis lumbriciformis	independent
Syngnathus rostellatus	independent
Syngnathus typhle	independent
Trachyrhamphus longirostris	independent
Hippocampus barbouri	independent
Pegasidae	
Eurypegasus draconis	independent
**Gobiomorpharia**	
Gobioidei	
Eleotridae	
Tateurndina ocellicauda	conjugated
Butidae	
Oxyeleotris marmorata	conjugated
Oxudercidae	
Trypauchen vagina	conjugated
Sicyopterus macrostetholepis	conjugated
Brachygobius doriae	conjugated
Chlamydogobius eremius	conjugated
Rhinogobius cliffordpopei	conjugated
Gobiidae	
Cryptocentrus cinctus	conjugated
Tomiyamichthys oni	conjugated
Paragobiodon xanthosoma	conjugated
Valenciennea strigata	conjugated
**Anabantomorpharia**	
Synbranchiformes	
Mastacembelidae	
Macrognathus pancalus	conjugated
Anabantiformes	
Helostomatidae	
Helostoma temminckii	conjugated
Osphronemidae	
Betta splendens	conjugated
Trichogaster chuna	conjugated
Trichopodus leerii	conjugated
Channidae	
Channa pulchra	conjugated
**Carangimorpharia**	
Carangiformes	
Rachycentridae	
Rachycentron canadum	conjugated
**Ovalentaria**	
Ovalentaria_i.s.	
Opistognathidae	
Opistognathus randalli	
Polycentridae	
Monocirrhus polyacanthus	conjugated
Pomacentridae	
Premnas biaculeatus	conjugated

Amphiprion ephippium	
Chromis retrofasciata	conjugated
Abudefduf saxatilis	
Amblyglyphidodon curacao	
Chrysiptera taupou	
Neoglyphidodon melas	
**Cichliformes**	
**Cichlidae**	
Astronotus ocellatus	conjugated
Aequidens pulcher	conjugated
Heros efasciatus	conjugated
Heros severus	conjugated
Pterophyllum scalare	conjugated
Thorichthys meeki	conjugated
Apistogramma agassizii	conjugated
Mikrogeophagus ramirezi	conjugated
Maylandia zebra	conjugated
Julidochromis marlieri	conjugated
Neolamprologus brichardi	conjugated
Steatocranus casuarius	conjugated
**Atheriniformes**	
**Melanotaeniidae**	
Glossolepis incisus	conjugated
Melanotaenia boesemani	conjugated
**Beloniformes**	
**Zenarchopteridae**	
Nomorhamphus ebrardtii	conjugated
**Cyprinodontiformes**	
**Poeciliidae**	
Heterandria formosa	conjugated
Poecilia	conjugated
Xiphophorus hellerii	conjugated
Xiphophorus maculatus	conjugated
**Goodeidae**	
Ameca splendens	conjugated
**Mugiliformes**	
**Mugilidae**	
Mugil cephalus	conjugated
**Gobiesociformes**	
**Gobiesocidae**	
Diademichthys lineatus	independent
**Blenniiformes**	
**Tripterygiidae**	
Helcogramma maldivensis	
**Blenniidae**	
Atrosalarias fuscus	independent
Blenniella chrysospilos	independent
Cirripectes stigmaticus	independent
Ecsenius stigmatura	independent
Exallias brevis	independent
Hypsoblennius brevipinnis	
Lipophrys pholis	conjugated
Meiacanthus grammistes	conjugated
Plagiotremus rhinorhynchos	
Salarias segmentatus	independent
**Percomorpharia**	
**Eupercaria_i.s.**	
**Pomacanthidae**	
Centropyge bicolor	

Centropyge loriculus	
Centropyge potteri	
Chaetodontoplus mesoleucus	
**Haemulidae**	
Plectorhinchus chaetodonoides	conjugated
**Lutjanidae**	
Lutjanus synagris	conjugated
**Uranoscopiformes**	
**Ammodytidae**	
Ammodytes tobianus	
**Labriformes**	
**Labridae**	
Hologymnosus doliatus	independent
Labroides dimidiatus	
Pseudocheilinus hexataenia	independent
Stethojulis bandanensis	
**Chaetodontiformes**	
**Chaetodontidae**	
Chaetodon auriga	
Hemitaurichthys zoster	
**Acanthuriformes**	
**Acanthuridae**	
Naso vlamingii	conjugated
Acanthurus bahianus	conjugated
Ctenochaetus truncatus	conjugated
**Zanclidae**	
Zanclus cornutus	
**Lophiiformes**	
**Antennarioidei**	
**Antennariidae**	
Antennarius .sp	conjugated
**Tetraodontiformes**	
**Tetraodontoidei**	
**Tetraodontidae**	
Arothron nigropunctatus	
Canthigaster valentini	independent
Carinotetraodon travancoricus	
Colomesus asellus	independent
**Balistoidei**	
**Balistidae**	
Balistapus undulatus	independent
Odonus niger	independent
Rhinecanthus aculeatus	independent
**Monacanthidae**	
Acreichthys tomentosus	independent
Oxymonacanthus longirostris	independent
Pervagor janthinosoma	independent
**Centrarchiformes**	
**Centrarchoidei**	
**Centrarchidae**	
Lepomis gibbosus	
**Cirrhitoidei**	
**Cirrhitidae**	
Amblycirrhitus pinos	independent
Cirrhitichthys aprinus	independent
Oxycirrhites typus	independent
Paracirrhites arcatus	independent
**Terapontoidei**	
**Kuhliidae**	

Kuhlia mugil	conjugated
**Perciformes**	
**Serranoidei**	
**Serranidae**	
Cephalopholis miniata	independent
Pseudanthias dispar	
**Percoidei**	
**Percidae**	
Perca fluviatilis	conjugated
Sander lucioperca	conjugated
**Scorpaenoidei**	
**Scorpaenidae**	
Dendrochirus zebra	conjugated
Ablabys taenianotus	conjugated
**Cottoidei**	
**Zoarcales**	
**Stichaeidae**	
Anoplarchus purpurescens	
Xiphister mucosus	
**Gasterosteales**	
**Gasterosteidae**	
Gasterosteus aculeatus	conjugated
**Cottales**	
**Cottidae**	
Cottus gobio	conjugated
Taurulus bubalis	conjugated

Suppl. Table 2: Literature survey on the presence of a fovea in ray finned fish. Species with a fovea (FO) are highlighted with green. All others have no fovea. Some reports simply state that a foves does not exists (no FO) or they classify the retinal distribution in regional differences (RD), Area Centralis (AC), and/or Horizontal streak (HS).

Species	Reference	Retina
**Actinopterygii**		
**Chondrostei**		
**Acipenseridae**		
Acipenser medirostris	Sillman et al. 2005	no FO
Scaphirhynchus albus	Sillman et al. 2005	no FO
**Polyodontidae**		
Polyodon spathula	Sillman and Dahlin 2004	no FO
**Lepisosteiformes**		
**Lepisosteidae**		
Lepisosteus platyrhincus	Collin and Northcutt 1993	AC-HS
**Teleost**		
**Elopomorpha**		
**Anguilliformes**		
**Synaphobranchidae**		
Synaphobranchus kaupii	Collin and Partridge 1996	RD
**Serrivomeridae**		
Serrivomer beanii	Collin and Partridge 1996	RD
**Clupeiformes**		
**Engraulidae**		
Engraulis mordax	O'Connell 1963	AC
Anchoa compressa	O'Connell 1963	AC
**Clupeidae**		
Sardina pilchardus	Marquez Legorreta 2019	RD
Sardinella aurita	Salem 2016	no FO
Sardinops sagax	O'Connell 1963	AC
Alosa sapidissima	O'Connell 1963	AC
**Alepocephaliformes**		
**Platytroctidae**		
Platytroctes apus	Locket 1971	FO
Searsia koefoedi	Collin and Partridge 1996	FO
Platytroctes apus	Collin and Partridge 1996	FO
**Alepocephalidae**		
Conocara macropterum	Collin et al. 2000	FO
Conocara murrayi	Collin et al. 2000	FO
Bajacalifornia drakei	Locket 1985	FO
Xenodermichthys copei	Collin and Partridge 1996	FO
Bathytroctes microlepis	Collin and Partridge 1996	FO
Alepocephalus rostratus	Collin and Partridge 1996	FO
Rouleina attrita	Collin and Partridge 1996	FO
Conocara murrayi	Collin and Partridge 1996	FO
**Cypriniformes**		
**Cyprinidae**		
Carassius auratus	Mednick and Springer 1988	AC
Aspius aspius	Zaunreiter et al. 1991	RD
Rutilus rutilus	Zaunreiter et al. 1991	RD
Abramis brama	Zaunreiter et al. 1991	RD

Cyprinus carpio	Zaunreiter et al. 1991	RD
Pelecus cultratus	Zaunreiter et al. 1991	RD
Garra gotyla	Nag and Bhattacharjee 2002	no FO
Garra lamta	Nag and Bhattacharjee 2002	no FO
Barilius bendelisis	Nag and Bhattacharjee 2002	no FO
Barilius vagra	Nag and Bhattacharjee 2002	no FO
Schizothorax richardsonii	Nag and Bhattacharjee 2002	no FO
Neolissochilus hexagonolepis	Nag and Bhattacharjee 2002	no FO
Danio aequipinnatus	Nag and Bhattacharjee 2002	no FO
Notemigonus crysoleucas	Kim and Park 2017	RD
Rutilus rutilus	Engström 1960	no FO
Scardinius erythrophthalmus	Engström 1960	no FO
Leuciscus idus	Engström 1960	no FO
Abramis brama	Engström 1960	no FO
Blicca bjoerkna	Engström 1960	no FO
Vimba vimba	Engström 1960	no FO
Cyprinus carpio	Engström 1960	no FO
Carassius carassius	Engström 1960	no FO
Carassius auratus	Engström 1960	no FO
Tinca tinca	Engström 1960	no FO
Danio rerio	Engström 1960	no FO
Phoxinus phoxinus	Engström 1960	no FO
Alburnus alburnus	Engström 1960	no FO
Exoglossum maxillingua	Collin and Ali 1994	AC
Semotilus atromaculatus	Collin and Ali 1994	AC
**Balitoridae**		
Balitora brucei	Nag and Bhattacharjee 2002	no FO
**Nemacheilidae**		
Nemacheilus beavani	Nag and Bhattacharjee 2002	no FO
Nemacheilus devdevi	Nag and Bhattacharjee 2002	no FO
**Siluriformes**		
**Ictaluridae**		
Ictalurus punctatus	Dunn-Meynell and Sharma 1987	HS
**Loricariidae**		
Liposcarus pardalis	Douglas et al. 2002	AC
**Amblycipitidae**		
Liobagrus mediadiposalis	Nag and Bhattacharjee 2002	no
**Gymnotiformes**		
**Apteronotidae**		
Apteronotus albifrons	Takiyama et al. 2015	RD
**Protacanthopterygii**		
**Salmonidae**		
Salmo salar	Ahlbert 1976	RD
Salmo trutta	Ahlbert 1976	RD
Salmo trutta	Anadón et al. 2001	no FO
Oncorhynchus keta	Beaudet et al. 1997	RD
Oncorhynchus tschawytscha	Beaudet et al. 1997	RD
Oncorhynchus kisutch	Beaudet et al. 1997	RD
Oncorhynchus mykiss	Beaudet et al. 1997	RD
**Argentiniformes**		
**Argentinidae**		
Argentina semifasciata	Tamura 1957	RD
**Stomiiformes**		

Sternoptychidae		
Argyropelecus sladeni	Collin and Partridge 1996	AC
Argyropelecus aculeatus	Collin and Partridge 1996	AC
Osmeriformes		
Osmeridae		
Osmerus eperlanus	Reckel et al. 2003	RD
Aulopiformes		
Scopelarchidae		
Scopelarchus michaelsarsi	Collin and Partridge 1996	AC
Ipnopidae		
Bathypterois dubius	Collin and Partridge 1996	AC
Chlorophthalmidae		
Chlorophthalmus albatrossis	Tamura 1957	RD
Notosudidae		
Scopelosaurus lepidus	Munk 1977	FO
Myctophiformes		
Myctophidae		
Myctophum punctatum	Collin and Partridge 1996	RD
Lampanyctus macdonaldi	Collin and Partridge 1996	RD
Paracanthopterygii		
Zeiformes		
Zeniontidae		
Zenion japonicum	Tamura 1957	RD
Gadiformes		
Merlucciidae		
Merluccius merluccius	Bozzano and Catalán 2002	AC
**Acanthopterygii**		
Holocentriformes		
Holocentridae		
Myripristis violacea	de Busserolles et al. 2020	AC
Neoniphon sammara	de Busserolles et al. 2020	AC
Sargocentron diadema	de Busserolles et al. 2020	AC
Batrachoidiformes		
Batrachoididae		
Halophryne diemensis	Collin and Pettigrew 1988	AC
Halophryne diemensis	Collin and Pettigrew 1989	AC
**Gobiaria**		
Kurtiformes		
Apogonidae		
Apogon lineatus	Tamura 1957	RD
Gobiiformes		
Oxudercidae		
Acanthogobius flavimanus	Miyazaki et al. 2019	AC
Gobiidae		
Zosterisessor ophiocephalus	Ota et al. 1999	AC
Scombriformes		
Scombridae		
Scomber japonicus	Tamura 1957	RD
Thunnus orientalis	Miyazaki 2013	AC
Scomber japonicus	O'Connell 1963	RD
Syngnathiformes		
Aulostomidae		
Aulostomus chinensis	Collin and Pettigrew 1988	AC-HS

Aulostomus chinensis	Collin and Pettigrew 1989	AC
**Syngnathidae**		
Corythoichthys paxtoni	Collin 19999	FO
Hippocampus .sp	Walls 1942	FO
Syngnathus typhle	Walls 1942	FO
Syngnathus acus	Walls 1942	FO
Syngnathus tenuirostris	Walls 1942	FO
Trachyrhamphus bicoarctatus	Easter 1992	FO
**Carangaria**		
**Carangaria_Incerta_sedis**		
**Toxotidae**		
Toxotes chatareus	Temple et al. 2013	RD
Toxotes .sp	Kahmann 1936	no FO
**Carangiformes**		
**Carangidae**		
Seriola quinqueradiata	Tamura 1957	RD
Trachurus japonicus	Tamura 1957	RD
Trachurus symmetricus	O'Connell 1963	RD
**Ovalentaria**		
**Ovalentaria_Incerta_sedis**		
**Pomacentridae**		
Amblyglyphidodon curacao	Collin and Pettigrew 1988	AC
Amblyglyphidodon curacao	Collin and Pettigrew 1989	AC
Amphiprion akindynos	Stieb et al. 2019	AC-HS
**Cichliformes**		
**Cichlidae**		
Nannacara anomala	Wagner 1974	RD
Nannacara anomala	Wagner 1974	AC
Maylandia zebra	Dalton et al. 2016	AC
**Atheriniformes**		
**Melanotaeniidae**		
Melanotaenia maccullochi	Reckel and Melzer 2003	RD
Glossolepis incisus	Reckel and Melzer 2003	RD
Marosatherina ladigesi	Reckel and Melzer 2003	RD
**Atherinidae**		
Atherina boyeri	Reckel and Melzer 2003	RD
**Cyprinodontiformes**		
**Goodeidae**		
Ameca splendens	Reckel and Melzer 2003	RD
**Fundulidae**		
Fundulus heteroclitus	Butcher 1938	AC
**Anablepidae**		
Anableps anableps	Oliveira et al. 2006	HS
Anableps microlepis	Schwassmann and Kruger 1965	HS
**Beloniformes**		
**Adrianichthyidae**		
Oryzias celebensis	Reckel and Melzer 2003	RD
**Exocoetidae**		
Parexocoetus mento	Reckel and Melzer 2003	RD
**Hemiramphidae**		
Hyporhamphus affinis	Reckel and Melzer 2003	RD
**Zenarchopteridae**		
Dermogenys pusilla	Reckel and Melzer 2003	RD

**Belonidae**		
Belone belone	Reckel et al. 2002	RD
Xenentodon cancila	Reckel and Melzer 2003	RD
Belone belone	Reckel and Melzer 2003	RD
Tylosurus crocodilus	Reckel and Melzer 2003	RD
**Scomberesocidae**		
Scomberesox saurus	Reckel and Melzer 2003	RD
Cololabis saira	Kondrashev and Gnyubkina 2011	FO
**Blenniiformes**		
**Tripterygiidae**		
Tripterygion delaisi	Fritsch et al. 2017	FO
Tripterygion delaisi	Ahlbert 1976	FO
Tripterygion delaisi	Neiße et al. 2020	FO
**Blenniidae**		
Salaria basilisca	Walls 1942	FO
Parablennius gattorugine	Walls 1942	FO
Parablennius sanguinolentus	Walls 1942	FO
Parablennius tentacularis	Walls 1942	FO
Blennius ocellaris	Walls 1942	FO
Salaria pavo	Walls 1942	FO
Petroscirtes variabilis	Collin and Pettigrew 1989	AC
Istiblennius edentulus	Easter 1992	FO
Parablennius gattorugine	Kahmann 1936	FO
Blennius ocellaris	Kahmann 1936	FO
Parablennius sanguinolentus	Kahmann 1936	FO
Parablennius tentacularis	Kahmann 1936	FO
**Eupercaria**		
**Eupercaria_Incerta_sedis**		
**Pomacanthidae**		
Pomacanthus semicirculatus	Collin and Pettigrew 1988	AC
Pomacanthus semicirculatus	Collin and Pettigrew 1989	AC
**Priacanthidae**		
Heteropriacanthus cruentatus	Tamura 1957	RD
**Uranoscopiformes**		
**Pinguipedidae**		
Parapercis cylindrica	Collin and Pettigrew 1988	AC
Parapercis cylindrica	Collin and Pettigrew 1989	AC
Parapercis nebulosa	Easter 1992	FO
Parapercis cylindrica	Easter 1992	FO
**Labriformes**		
**Labridae**		
Choerodon cyanodus	Collin and Pettigrew 1988	AC-HS
Coris julis	Walls 1942	FO
Choerodon cyanodus	Collin and Pettigrew 1989	AC
Thalassoma pavo	Kahmann 1936	FO
Thalassoma lunare	Kahmann 1936	FO
Coris julis	Kahmann 1936	FO
Labrus .sp	Kahmann 1936	AC
Symphodus .sp	Kahmann 1936	AC
**Chaetodontiformes**		
**Leiognathidae**		
Leiognathus equula	Tamura 1957	RD
**Acanthuriformes**		

**Acanthuridae**		
Prionurus scalprum	Tamura 1957	RD
**Spariformes**		
**Lethrinidae**		
Lethrinus miniatus	Collin and Pettigrew 1988	AC-HS
Gymnocranius audleyi	Collin and Pettigrew 1988	AC-HS
Lethrinus miniatus	Collin and Pettigrew 1989	AC
Gymnocranius audleyi	Collin and Pettigrew 1989	AC
**Sparidae**		
Acanthopagrus butcheri	Shand et al. 2000	AC
Acanthopagrus butcheri	Shand et al. 2000	AC
Pagrus major	Tamura 1957	RD
Acanthopagrus berda	Tamura 1957	RD
Acanthopagrus schlegelii	Tamura 1957	RD
Dentex tumifrons	Tamura 1957	RD
**Tetraodontiformes**		
**Ostraciidae**		
Lactoria cornuta	Kahmann 1936	FO
**Balistidae**		
Balistoides conspicillum	Collin and Pettigrew 1988	AC-HS
Balistoides conspicillum	Collin and Pettigrew 1989	AC
Rhinecanthus aculeatus	Champ et al. 2014	AC
Balistes capriscus	Kahmann 1936	FO
Rhinecanthus aculeatus	Kahmann 1936	FO
**Monacanthidae**		
Navodon modestus	Ito and Murakami 1984	AC
Thamnaconus modestus	Tamura 1957	RD
**Molidae**		
Mola mola	Kino et al. 2009	RD
**Tetraodontidae**		
Marilyna pleurosticta	Collin and Pettigrew 1989	AC
Takifugu chrysops	Tamura 1957	RD
Takifugu niphobles	Tamura 1957	RD
Canthigaster valentini	Easter 1992	FO
Tetraodon fluviatilis	Kahmann 1936	FO
**Pempheriformes**		
**Acropomatidae**		
Malakichthys wakiyae	Tamura 1957	RD
**Creediidae**		
Limnichthys fasciatus	Collin and Collin 1988	FO
**Howellidae**		
Howella sherborni	Collin and Partridge 1996	FO
**Lateolabracidae**		
Lateolabrax japonicus	Tamura 1957	RD
**Centrarchiformes**		
**Centrarchidae**		
Lepomis macrochirus	Gomi and Miyazaki 2015	AC
Lepomis cyanellus	Cameron 1995	AC
**Cirrhitidae**		
Cirrhitichthys falco	Easter 1992	FO
**Kyphosidae**		
Girella .sp	Walls 1942	FO
Girella punctata	Tamura 1957	RD

**Terapontidae**		
Rhynchopelates oxyrhynchus	Tamura 1957	RD
**Perciformes**		
**Serranoidei**		
**Serranidae**		
Cephalopholis miniata	Collin and Pettigrew 1988	AC
Serranus cabrilla	Walls 1942	FO
Serranus hepatus	Walls 1942	FO
Serranus scriba	Walls 1942	FO
Paralabrax clathratus	Schwassmann 1968	FO
Paralabrax maculatofasciatus	Schwassmann 1968	FO
Paralabrax nebulifer	Schwassmann 1968	FO
Cephalopholis miniata	Collin and Pettigrew 1989	AC
Hyporthodus septemfasciatus	Tamura 1957	RD
Epinephelus chlorostigma	Tamura 1957	RD
Serranus hepatus	Kahmann 1936	FO
**Percoidei**		
**Percidae**		
Perca fluviatilis	Guthrie and Banks 1978	RD
**Trachinidae**		
Trachinus draco	Kahmann 1936	FO
Echiichthys vipera	Kahmann 1936	FO
**Nothothenioidei**		
**Channichthyidae**		
Champsocephalus gunnari	Miyazaki et al. 2011	AC
**Scorpaenoidei**		
**Sebastidae**		
Sebastiscus marmoratus	Ito and Murakami 1984	AC
Sebastiscus marmoratus	Tamura 1957	RD
Helicolenus dactylopterus	Tamura 1957	RD
**Triglioidei**		
**Triglidae**		
Chelidonichthys kumu	Tamura 1957	RD
**Cottoidei**		
**Agonidae**		
Agonus cataphractus	Kahmann 1936	FO
**Cottidae**		
Pseudoblennius totomius	Tamura 1957	RD
**Pholidae**		
Pholis gunellus	Walls 1942	FO
**Zoarcidae**		
Zoarces .sp	Kahmann 1936	AC

References

Ahlbert, I.-B. (1976) Organization of the cone cells in the retinae of salmon (*Salmo salar*) and trout (*Salmo trutta trutta*) in relation to their feeding habits. *Acta Zoologica* 57:13-35. DOI:10.1111/j.1463-6395.1976.tb00208.x

Anadón, R., Becerra, M., Díaz, M.L., Manso, M.J. (2001). Presence and development of thyrotropin-releasing hormone-immunoreactive amacrine cells in the retina of a teleost, the brown trout (*Salmo trutta fario*). *Neurosci. Lett.* 299:225-228. DOI:10.1016/s0304-3940(01)01531-2

Beaudet, L., Flamarique, I.N., Hawryshyn, C.W. (1997). Cone photoreceptor topography in the retina of sexually mature Pacific salmonid fishes. *The Journal of Comparative Neurology* 383:49-59. DOI:10.1002/(SICI)1096-9861(19970623)383:1<49::AID-CNE4>3.0.CO,2-L

Bozzano, A., Catalán, I.A. (2002). Ontogenetic changes in the retinal topography of the european hake, *Merluccius merluccius* : implications for feeding and depth distribution. *Mar. Biol.* 141:549-559. DOI:10.1007/s00227-002-0840-7

Butcher, E.O. (1938). The structure of the retina of *Fundulus heteroclitus* and the regions of the retina associated with the different chromatophoric responses. *J. exp. Zool.* 79:275-297. DOI:10.1002/jez.1400790207

Cameron, D.A. (1995). Asymmetric retinal growth in the adult teleost green sunfish (*Lepomis cyanellus*). *Vis. Neurosci.* 12:95-102. DOI:10.1017/s0952523800007343

Champ, C., Wallis, G., Vorobyev, M., Siebeck, U., Marshall, J. (2014). Visual acuity in a species of coral reef fish: *Rhinecanthus aculeatus*. *Brain Behav. Evol.* 83:31-42. DOI:10.1159/000356977

Collin, S.P., Partridge, J.C. (1996). Retinal specializations in the eyes of deep-sea teleosts. *Journal of Fish Biology* 49:157-174. DOI:10.1111/j.1095-8649.1996.tb06073.x

Collin, S.P., Lloyd, D.J., Wagner, H.J. (2000). Foveate vision in deep–sea teleosts: a comparison of primary visual and olfactory inputs. *Philosophical Transactions of the Royal Society of London. Series B: Biological Sciences* 355:1315-1320. DOI:10.1098/rstb.2000.0691

Collin, S.P., Pettigrew, J.D. (1988). Retinal Topography in Reef Teleosts. I. Some species with well-developed areae but poorly-developed streaks. *Brain, Behavior and Evolution* 31:269-282. DOI:10.1159/000116594

Collin, S.P., Pettigrew, J.D. (1989). Quantitative comparison of the limits on visual spatial resolution set by the ganglion cell layer in twelve species of reef Teleosts. *Brain, Behavior and Evolution* 34:184-192. DOI:10.1159/000116504

Collin, S.P., Pettigrew, J.D. (1988). Retinal topography in reef Teleosts. II. Some species with prominent horizontal streaks and high-density areae. *Brain, Behavior and Evolution* 31:283-295. DOI:10.1159/000116595

Collin, S.H. (19999) The foveal photoreceptor mosaic in the pipefish, *Corythoichthyes paxtoni* (Syngnathidae, Teleostei). *Histol. Histopathol.* 14:369-382. DOI:10.14670/HH-14.369.

Collin, S.P., Northcutt, R.G (1993) The visual system of the florida garfish, *Lepisosteus platyrhincus* (Ginglymodi). iii. retinal ganglion cells. *Brain Behav. Evol.* 42:295-320. DOI:10.1159/000114168

Collin, S P., Ali, M.A (1994) Multiple areas of acute vision in two freshwater teleosts, the creek chub, *Semotilus atromaculatus* (mitchill) and the cutlips minnow, exoglossum maxillingua (lesueur). *Can. J. Zool.* 72:721-730. DOI:10.1139/z94-097

Collin, S P., Collin, H.B (1988) Topographic analysis of the retinal ganglion cell layer and optic nerve in the sandlance *Limnichthyes fasciatus* (Creeiidae, Perciformes). *J. Comp. Neurol.* 278:226-241. DOI:10.1002/cne.902780206

Dalton, B.E., de Busserolles, F., Marshall, N.J., Carleton, K.L. (2016). Retinal specialization through spatially varying cell densities and opsin coexpression in cichlid fish. *Journal of Experimental Biology* 220:266-277. DOI:10.1242/jeb.149211

de Busserolles, F., Cortesi, F., Fogg, L., Stieb, S.M., Luehrmann, M., Marshall, N. J. (2020). The visual ecology of Holocentridae, a nocturnal coral reef fish family with a deep-sea-like multibank retina. *Journal of Experimental Biology* 224:jeb233098. DOI:10.1242/jeb.233098

Douglas, R.H., Collin, S.P., Corrigan, J. (2002). The eyes of suckermouth armoured catfish (Loricariidae, subfamily Hypostomus): pupil response, lenticular longitudinal spherical aberration and retinal topography. *Journal of Experimental Biology* 205:3425-3433. DOI:10.1242/jeb.205.22.3425

Dunn-Meynell, A.A., Sharma, S.C. (1987). Visual system of the channel catfish (*Ictalurus punctatus*): ii. the morphology associated with the multiple optic papillae and retinal ganglion cell distribution. *J. Comp. Neurol.* 257:166-175. DOI:10.1002/cne.902570204

Easter, S.S. (1992). Retinal growth in foveated teleosts: nasotemporal asymmetry keeps the fovea in temporal retina. The *Journal of Neuroscience* 12:2381-2392. DOI:10.1523/JNEUROSCI.12-06-02381.1992

Engström, K. (1960). Cone types and cone arrangement in the retina of some cyprinids. *Acta Zoologica* 41:277-295. DOI:10.1111/j.1463-6395.1960.tb00481.x

Fritsch, R, Collin, S P., Michiels, N K. (2017) Anatomical analysis of the retinal specializations to a crypto-benthic, micro-predatory lifestyle in the Mediterranean Triplefin Blenny *Tripterygion delaisi*. *Frontiers in Neuroanatomy* 11:122. DOI:10.3389/fnana.2017.00122

Gomi, Y, Miyazaki, T. (2015). Transition of the retinal area centralis in bluegill *Lepomis macrochirus* as an implication of changes in feeding ecology with age. *Fish. Sci.* 81:673-678. DOI:10.1007/s12562-015-0880-9

Guthrie, D.M., Banks, J.R. (1978). The receptive field structure of visual cells from the optic tectum of the freshwater perch (*Perca fluviatilis*). *Brain Res.* 141:211-225. DOI:10.1016/0006-8993(78)90193-2

Ito, H., Murakami, T. (1984). Retinal ganglion cells in two teleost species, *Sebastiscus marmoratus* and *Navodon modestus*. *J. Comp. Neurol.* 229:80-96. DOI:10.1002/cne.902290107

Kahmann, H. (1936). Über das foveale Sehen der Wirbeltiere. *Albrecht von Græfes Archiv für Ophthalmologie* 135:265-276. DOI:10.1007/BF01856858

Kim, J.G., Park, J.Y. (2017). A Histological study on the visual cell layer of the endemic korean species *Liobagrus mediadiposalis* (Pisces: Amblycipitidae). *Applied Microscopy* 47:238-241. DOI:10.9729/AM.2017.47.4.238

Kino, M., Miayzaki, T., Iwami, T., Kohbara, J. (2009). Retinal topography of ganglion cells in immature ocean sunfish, *Mola mola*. *Environ. Biol. Fish.* 85:33-38. DOI:10.1007/s10641-009-9453-z

Kondrashev, S.L., Gnyubkina, V.P. (2011). Peculiarities of the eye morphology and the spectral sensitivity of the retinal photoreceptors of the pacific saury *Cololabis saira*. *Russ. J. Mar. Biol.* 37:143-150. DOI:10.1134/s1063074011020064

Locket, N.A. (1971). Retinal structure in *Platytroctes apus*, a deep-sea fish with a pure rod fovea. *J. Mar. Biol. Assoc. U. K.* 51:79. DOI:10.1017/s0025315400006470

Locket, N.A. (1985). The multiple bank rod fovea of *Bajacalifornia drakei*, an alepocephalid deep-sea teleost. *Proc. R. Soc. Lond., B, Biol. Sci.* 224:7-22. DOI:10.1098/rspb.1985.0018

Marquez Legorreta, E. (2019). Visual learning and its underlying neural substrate in two species of teleost fish (Zebrafish and Ambon damselfish). : University of Queensland Library. DOI:10.14264/uql.2020.647

Mednick, A.S., Springer, A.D. (1988). Asymmetric distribution of retinal ganglion cells in goldfish. *J. Comp. Neurol.* 268:49-59. DOI:10.1002/cne.902680106

Miyazaki, T., Kato, A., Ikenaga, T., Hagio, H., Yamamoto, N. (2019). A lambda-shaped retractor lentis muscle in the yellowfin goby *Acanthogobius flavimanus*. *Journal of Morphology* 280:526-533. DOI:10.1002/jmor.20961

Miyazaki, T., Iwami, T., Meyer-Rochow, V.B. (2011). The position of the retinal area centralis changes with age in *Champsocephalus gunnari* (channichthyidae), a predatory fish from coastal antarctic waters. *Polar Biol.* 34:1117-1123. DOI:10.1007/s00300-011-0969-2

Miyazaki, T. (2013). Retinal ganglion cell topography in juvenile pacific bluefin tuna *Thunnus orientalis* (Temminck and schlegel). *Fish Physiol. Biochem.* 40:23-32. DOI:10.1007/s10695-013-9820-8

Munk, O. (1977). The visual cells and retinal tapetum of the foveate deep-sea fish *Scopelosaurus lepidus* (Teleostei). *Zoomorphologie* 87:21-49. DOI:10.1007/bf02568740

Nag, T.C., Bhattacharjee, J. (2002). Retinal cytoarchitecture in Some Mountain-stream Teleosts of India. *Environmental Biology of Fishes* 63:435-449. DOI:10.1023/A:1014982218347

Neiße, N., Santon, M., Bitton, P.P., Michiels, N.K. (2020). Small benthic fish strike at prey over distances that fall within theoretical predictions for active sensing using light. *Journal of Fish Biology* 97:1201-1208. DOI:10.1111/jfb.14502

O'Connell, C.P. (1963). The structure of the eye ofsardinops caerulea, *Engraulis mordax*, and four other pelagic marine teleosts. *J. Morphol.* 113:287-329. DOI:10.1002/jmor.1051130214

Oliveira, F.G., Coimbra, J.P., Yamada, E.S., Montag, L.F.D.A., Nascimento, F.L., Oliveira, V.A., Mota, D.L.D., Bittencourt, A.M., Silva, V.L.D., Costa, B.L.D.S.A.D. (2006). Topographic analysis of the ganglion cell layer in the retina of the four-eyed fish *Anableps anableps*. *Vis. Neurosci.* 23:879-886. DOI:10.1017/s0952523806230232

Ota, D., Francese, M., Ferrero, E.A. (1999). Vision in the grass goby, *Zosterisessor ophiocephalus* (Teleostei, Gobiidae): a morphological and behavioural study. *Ital. J. Zool.* 66:125-139. DOI:10.1080/11250009909356247

Reckel, F., Hoffmann, B., Melzer, R.R., Horppila, J., Smola, U. (2003). Photoreceptors and cone patterns in the retina of the smelt *Osmerus eperlanus* (l.) (Osmeridae: Teleostei). *Acta Zoologica* 84:161-170. DOI:10.1046/j.1463-6395.2003.00142.x

Reckel, F., Melzer, R.R. (2003). Regional variations in the outer retina of Atherinomorpha (Beloniformes, Atheriniformes, Cyprinodontiformes: Teleostei): photoreceptors, cone patterns, and cone densities. *J. Morphol.* 257:270-288. DOI:10.1002/jmor.10122

Reckel, F., Melzer, R.R., Smola, U. (2002). Outer retinal fine structure of the garfish *Belone belone* (l.) (Belonidae, Teleostei) during light and dark adaptation - photoreceptors, cone patterns and densities. *Acta Zoologica* 82:89-105. DOI:10.1046/j.1463-6395.2001.00071.x

Salem, M.A. (2016). Structure and function of the retinal pigment epithelium, photoreceptors and cornea in the eye of *Sardinella aurita* (Clupeidae, Teleostei). T*he Journal of Basic & Applied Zoology* 75:1-12. DOI:10.1016/j.jobaz.2015.12.001

Schwassmann, H.O. (1968). Visual projection upon the optic tectum in foveate marine teleosts. *Vision Research* 8:1337-1348. DOI:10.1016/0042-6989(68)90054-0

Schwassmann, H.O., Kruger, L. (1965). Experimental analysis of the visual system of the four-eyed fish *Anableps microlepis*. *Vision Res.* 5:269-281. DOI:10.1016/0042-6989(65)90004-0

Shand, J., Chin, S.M., Harman, A.M., Collin, S.P. (2000). The relationship between the position of the retinal area centralis and feeding behaviour in juvenile black bream *Acanthopagrus butcheri* (sparidae: Teleostei). *Philos. Trans. R. Soc. Lond. B Biol. Sci.* 355:1183-1186. DOI:10.1098/rstb.2000.0663

Shand, J., Chin, S.M., Harman, A.M., Moore, S., Collin, S.P. (2000). Variability in the location of the retinal ganglion cell area centralis is correlated with ontogenetic changes in feeding behavior in the black bream, *Acanthopagrus butcheri* (Sparidae, Teleostei). *Brain Behav. Evol.* 55:176-190. DOI:10.1159/000006651

Sillman, A.J., Dahlin, D.A. (2004). Photoreceptor topography the paddlefish in the duplex retina of the paddlefish (*Polyodon spathula*). *J. Exp. Zool. Comp. Exp. Biol.* 301A:674-681. DOI:10.1002/jez.a.63

Sillman, A.J., Beach, A.K., Dahlin, D.A., Loew, E.R. (2005). Photoreceptors and visual pigments in the retina of the fully anadromous green sturgeon (*Acipenser medirostrus*) and the potamodromous pallid sturgeon (*Scaphirhynchus albus*). *J. Comp. Physiol.* A 191:799-811. DOI:10.1007/s00359-005-0004-6

Stieb, S.M., de Busserolles, F., Carleton, K.L., Cortesi, F., Chung, W.-S., Dalton, B.E., Hammond, L.A., Marshall, N. J. (2019). A detailed investigation of the visual system and visual ecology of the Barrier Reef anemonefish, *Amphiprion akindynos*. *Scientific Reports 9*:16459. DOI:10.1038/s41598-019-52297-0

Takiyama, T., Luna Da Silva, V., Moura Silva., D, Hamasaki, S., Yoshida, M. (2015). Visual capability of the weakly electric fish *Apteronotus albifrons* as revealed by a modified retinal flat-mount method. *Brain Behav. Evol.* 86:122-130. DOI:10.1159/000438448

Tamura, T. (1957). A study of visual perception in fish, especially on resolving power and accommodation. *Bulletin of the Japanese Society of Scientific Fisheries* 22:536-557. DOI:10.2331/suisan.22.536

Temple, S.E., Manietta, D., Collin, S.P. (2013). A comparison of behavioural (landolt c) and anatomical estimates of visual acuity in archerfish (*Toxotes chatareus*). *Vision Res.* 83:1-8. DOI:10.1016/j.visres.2013.02.014

Wagner, H.-J. (1974). Die Entwicklung der Netzhaut von *Nannacara anomala* (Regan) (Cichlidae, Teleostei) mit besonderer Berücksichtigung regionaler Differenzierungsunterschiede. Z*. Morph. Tiere* 79:113-131. DOI:10.1007/bf00298778

Walls, G.L. (1942). The vertebrate eye and its adaptive radiation. : Hafner Pub. Co.

Zaunreiter, M., Junger, H., Kotrschal, K. (1991). Retinal morphology of cyprinid fishes: a quantitative histological study of ontogenetic changes and interspecific variation. *Vision Res.* 31:383-394. DOI:10.1016/0042-6989(91)90091-i
